# OA10 Pregnancy-associated osteoporosis - a rare subset of bone disease

**DOI:** 10.1093/rap/rkad070.010

**Published:** 2023-09-27

**Authors:** Zhongyang Xia, Rizwan Rajak

**Affiliations:** Croydon University Hospital, London, United Kingdom; Croydon University Hospital, London, United Kingdom

## Abstract

**Introduction:**

Pregnancy-associated osteoporosis is a rare but serious condition in which patients present with fragility fractures (often vertebral) in the last trimester of pregnancy or the early postpartum period, generally in the first twelve weeks. The underlying pathological process is not yet clear – various causative mechanisms have been proposed, and it is possible that there is significant heterogeneity in aetiology. Research into this disease is limited by its rarity and diagnostic complexity, and at present there is no standard approach to management. It is essential to promote greater recognition and understanding so that this condition is identified early and managed appropriately.

**Case description:**

A 33-year-old lady developed acute back pain around 11 weeks post-partum following her first pregnancy. She had been breastfeeding. Her symptoms initially affected the right side and rib area, which progressed over the next month to the lower back.

MRI imaging revealed an acute crush fracture of the L2 vertebrae with 30% height loss as well as a non-acute moderate wedge fracture to the T11 vertebrae with 30% height loss.

Vitamin D, haematinics, bone profile, parathyroid hormone level and full blood count were normal, as well as thyroid, renal and liver function. Genetic testing for COL1A1/2 was negative.

Bone density scan showed severe osteoporosis in the lumbar spine (mean Z-score –3.5) and osteopenia in the left hip (total hip score –1.7, femoral neck score –1.8).

The patient had a normal BMI (19.8) and age of menarche (13 years old). She was on the oral contraceptive pill for menorrhagia until 29 years old. No family history of osteoporosis or parental hip fracture was reported. Her regular medications included sertraline. There was no history of eating disorder, and she had reasonable dietary dairy intake. The patient was an ex-social smoker and drank less than 10 units of alcohol per week. With regards to family planning, she was keen to have more children in the future.

The patient was commenced on teriparatide and Osteocare supplement. A longer-term plan was made to re-assess bone density after the teriparatide course to decide whether anti-resorptive follow-on therapy was required. Buprenorphine patches and diazepam were provided, and she was referred for graded exercises with physiotherapy.

At 4-month follow-up, the patient reported good treatment compliance, with no side effects. She has not needed to use the diazepam or buprenorphine patches and symptomatically her back was much better. Repeat MRI imaging showed that the vertebral fractures were healing well.

**Discussion:**

The disease mechanism behind pregnancy-associated osteoporosis has not been clearly determined. Research using high resolution CT imaging has demonstrated significant trabecular microarchitectural weaknesses in patients when compared to healthy lactating control subjects. This finding corresponds with the clinical presentation of the condition with fractures in trabecular bones. In many patients, no apparent causative factor is found, but some patients have been identified to have risk factors for low bone density (either before or during the pregnancy) and family history of osteoporotic fractures.

Normal pregnancy can be associated with bone losses of around 3-5% at the spine and hip. There is also strong evidence that lactation can lead to bone loss of 3-10% at the above sites as well, with the duration of lactation and amenorrhoea associated with the degree of bone loss. This is due to loss of calcium, fall in oestrogen level and rise in parathyroid hormone related protein (PTHrP) level. Therefore, patients who already have pre-existing low bone mineral density may develop fractures as a result of skeletal stress caused by pregnancy and lactation. It is thought that this bone loss gradually reverses during and after weaning, and the process of recovery may continue for around 18 months or possibly even longer, which has important implications for pharmacological management of this condition.

There may also be a hereditary element involved, as evidenced by familial investigations and genetic panel screening. This has identified several phenotypical variants which have been found to be associated with reduced bone turnover, and this was thought to be due to abnormal osteoblast function or other bone formation defects leading to increased bone resorption and reduced bone formation.

Further research is needed to increase our understanding of the development of this condition, which will aid with disease prevention as well as more effective therapeutic management.

**Key learning points:**

There remains a lack of clear consensus on the optimal treatment pathway for pregnancy-associated osteoporosis. The main pharmacological options at present include anti-resorptive therapy (bisphosphonates), anabolic therapy (teriparatide) and monoclonal antibody therapy (denosumab). Individually, these have been shown to increase bone mineral density with symptomatic improvement and reduction in further fracture risk, but there have been no high-quality studies directly comparing the efficacy of each treatment.

Bisphosphonates have been in use for decades, with a well-established evidence base and safety profile. Potential risks in long-term use include osteonecrosis of the jaw and atypical femoral fractures, but these are relatively rare. However, their long half-life and persistence in maternal bone and serum have limited their use in reproductive-age women, given the possibility of these medications passing the placental barrier in pregnancy and adversely affecting fetal health.

Teriparatide and denosumab are usually reserved for severe disease or contraindication/intolerance of other treatments, but there is a lack of long-term safety data for both agents. The course of teriparatide is generally limited to two years due to insufficient demonstration of efficacy beyond this timeframe, and it is unclear if subsequent consolidative therapy with another agent is required due to conflicting evidence on whether there is deterioration of bone density after teriparatide is discontinued.

There is still much we have to learn about the management of pregnancy-associated osteoporosis. Interesting research directions include comparison of individual therapies in randomised controlled trials, assessing the effect of combination therapy as well as looking into the long-term efficacy and safety profiles of teriparatide and denosumab. Furthermore, as there have been studies showing that there can be some recovery in bone density in this condition without treatment, further investigation into this area would be useful in clarifying the threshold for patients requiring pharmacological treatment as compared to conservative management.

